# Identification and validation of a novel four-gene diagnostic model for neonatal early-onset sepsis with bacterial infection

**DOI:** 10.1007/s00431-022-04753-9

**Published:** 2022-12-17

**Authors:** Yong Bai, Na Zhao, Zhenhua Zhang, Yangjie Jia, Genhao Zhang, Geng Dong

**Affiliations:** 1 Children’s Hospital Affiliated to Zhengzhou University, Zhengzhou Key Laboratory of Children’s Infection and Immunity, Zhengzhou, China; 2 Department of Pathology, Zhengzhou Central Hospital Affiliated to Zhengzhou University, Zhengzhou, China; 3 Department of Blood Transfusion, The First Affiliated Hospital of Zhengzhou University, Zhengzhou, China

**Keywords:** Neonatal, EOS, Diagnostic model, ROC curves, miRNA-mRNA network

## Abstract

Neonatal early-onset sepsis (EOS) has unfortunately been the third leading cause of neonatal death worldwide. The current study is aimed at discovering reliable biomarkers for the diagnosis of neonatal EOS through transcriptomic analysis of publicly available datasets. Whole blood mRNA expression profiling of neonatal EOS patients in the GSE25504 dataset was downloaded and analyzed. The binomial LASSO model was constructed to select genes that most accurately predicted neonatal EOS. Then, ROC curves were generated to assess the performance of the predictive features in differentiating between neonatal EOS and normal infants. Finally, the miRNA-mRNA network was established to explore the potential biological mechanisms of genes within the model. Four genes (CST7, CD3G, CD247, and ANKRD22) were identified that most accurately predicted neonatal EOS and were subsequently used to construct a diagnostic model. ROC analysis revealed that this diagnostic model performed well in differentiating between neonatal EOS and normal infants in both the GSE25504 dataset and our clinical cohort. Finally, the miRNA-mRNA network consisting of the four genes and potential target miRNAs was constructed. Through bioinformatics analysis, a diagnostic four-gene model that can accurately distinguish neonatal EOS in newborns with bacterial infection was constructed, which can be used as an auxiliary test for diagnosing neonatal EOS with bacterial infection in the future.

*Conclusion*: In the current study, we analyzed gene expression profiles of neonatal EOS patients from public databases to develop a genetic model for predicting sepsis, which could provide insight into early molecular changes and biological mechanisms of neonatal EOS.

**Table:** 

**What is Known:** *• Infants with suspected EOS usually receive empiric antibiotic therapy directly after birth.* *• When blood cultures are negative after 48 to 72 hours, empirical antibiotic treatment is often halted. Needless to say, this is not a short time. Additionally, because of the concern for inadequate clinical sepsis production and the limited sensitivity of blood cultures, the duration of antibiotic therapy for the kid is typically extended.*	
**What is New:** *• We established a 4-gene diagnostic model of neonatal EOS with bacterial infection by bioinformatics analysis method. The model has better diagnostic performance compared with conventional inflammatory indicators such as CRP, Hb, NEU%, and PCT.*

**What is Known:**

*• Infants with suspected EOS usually receive empiric antibiotic therapy directly after birth.*

*• When blood cultures are negative after 48 to 72 hours, empirical antibiotic treatment is often halted. Needless to say, this is not a short time. Additionally, because of the concern for inadequate clinical sepsis production and the limited sensitivity of blood cultures, the duration of antibiotic therapy for the kid is typically extended.*

**What is New:**

*• We established a 4-gene diagnostic model of neonatal EOS with bacterial infection by bioinformatics analysis method. The model has better diagnostic performance compared with conventional inflammatory indicators such as CRP, Hb, NEU%, and PCT.*

## Introduction

Early-onset sepsis (EOS) in newborns is a multiorgan system dysfunction that can lead to severe neonatal morbidity and mortality when pathogenic microbial strains are isolated from peripheral blood or cerebrospinal fluid within 7 days of birth [1]. Acute kidney injury (AKI) is one of the most common conditions presenting with neonatal EOS [2]. In practice, many newborns with suspected EOS are given intravenous broad-spectrum antibiotics for several days, which may interfere with early breastfeeding, lead to bacterial dysbiosis, and increase the risk of morbidity for many diseases, such as type I diabetes, asthma, and necrotizing enterocolitis (NEC), although the incidence of neonatal EOS is less than 6 per 1,0000 [3–6]. Early antibiotic treatment of very low birth weight preterm infants with suspected sepsis without blood cultures is associated with an increased risk of subsequent morbidity and mortality [7, 8]. In addition, no studies to date have shown that infants with EOS could benefit from antibiotic therapy. Due to the limited predictive power of routine laboratory tests such as complete blood count, erythrocyte sedimentation rate (ESR), procalcitonin (PCT), and C-reactive protein (CRP) to effectively distinguish neonatal EOS from suspected EOS, this may prolong the use of antibiotics in uninfected infants [9–11]. Hence, attempts to discover specific biomarkers of neonatal EOS and to reduce unnecessary antibiotic exposure in neonates with suspected sepsis are exceptionally important for clinical neonatal care. In the current study, we analyzed gene expression profiles of neonatal EOS patients from public databases to develop a genetic model for predicting sepsis, which could provide insight into early molecular changes and biological mechanisms of neonatal EOS.

## Materials and methods

### Obtainment of the GSE25504 cohort and identification of differentially expressed genes (DEGs)

Whole blood mRNA expression profiling of neonatal EOS patients in the GSE25504 dataset was downloaded from gene expression omnibus (GEO) databases (http://www.ncbi.nlm.nih.gov/geo/), which included microarray data from four platforms (GPL570, GPL6947, GPL13667, and GPL15158). Then, the Limma R package with cut-off criteria of *p* value less than 0.05 and |logFC|> 0.5 was used to identify DEGs between the sepsis and control groups on four platforms of the GSE25504 dataset. The overlapping DEGs among the four platforms were used to identify gene set enrichment with Gene set enrichment analysis (GSEA) in the Metascape database [12]. Results were considered statistically significant when the *p* value was less than 0.05.

### Construction of the least absolute shrink and selection operator (LASSO) model

Based on these overlapping DEGs, the binomial LASSO model was constructed using the glmnet package on the GPL6947 platform. LASSO is a more refined model obtained by constructing a penalty function that makes it compress some coefficients while setting some coefficients to zero. Therefore, it retains the advantage of subset shrinkage and is a kind of biased estimation dealing with data with complex covariance. The characteristic of LASSO regression is that when building a generalized linear model, variables can be selectively put into the model to obtain better performance parameters, thus avoiding overfitting. Here, the generalized linear model contains one-dimensional continuous dependent variables, multidimensional continuous dependent variables, nonnegative count dependent variables, binary discrete dependent variables, and multivariate discrete dependent variables. The complexity of LASSO is controlled by *λ*, and the larger *λ* penalizes linear models with more variables more strongly, so as to finally obtain a good model with fewer variables. The score was at the last setup based on the premise of directly combining the equation underneath with the mRNA expression level duplicating the LASSO regression coefficient (*β*) when *λ*_*min*_ was confirmed. *Score* = (*βmRNA1* × *mRNA1*) + (*βmRNA2* × *mRNA2*) + … + (*βmRNAn* × m*RNAn*). The accuracy of the predictive features was assessed by ROC examination. Receiver operating characteristic curve (ROC) analysis was used to examine the model’s ability to discriminate between EOS and normal infants.

### Clinical specimens and quantitative real-time PCR (qRT-PCR) analysis

In the period from 1 Jan 2022 to 30 Jun 2022, 99 normal and 60 EOS infants from Children’s Hospital Affiliated to Zhengzhou University were included in this single-center retrospective case–control study. EOS infants presented with significant clinical signs and symptoms of sepsis (respiratory distress, anemia, fever, decreased absolute neutrophil count, or increased C-reactive protein) were confirmed with the results of positive blood culture. Peripheral blood mononuclear cells (PBMCs) were isolated from 1 mL of whole blood collected from each infant by density separation on a Ficoll-Paque. After total RNA was extracted from PBMCs, qRT-PCR was used to detect the mRNA levels of genes in the model [13]. Finally, the relative mRNA expression levels were normalized to *β*-ACTIN. Primer sequences are shown in Table 1.

**Table 1:** The sequences of the qRT-PCR primers used in this study

Gene	Forward primer	Reverse primer
CST7	GTGTGAAGCCAGGATTTCCTAA	TGTCGTTCGTGCAGTTGTTGA
CD3G	TGGCCCAGTCAATCAAAGGAA	CAAGTCAGAAGTACCGAACCATC
CD247	GGCACAGTTGCCGATTACAGA	CTGCTGAACTTCACTCTCAGG
CD8B	AGACCCCTGCATACATAAAGGT	CGCTGTCTCAGCCAGTAGAT
SIRPG	CCCGGCATCATCCCTTACTG	TTCCAGGGGACGTAGATGGG
ANKRD22	AGGGCATGTGAGAATCGTTTC	GTAGCATTCGTACAAGAGCCTC
MAL	ACCGCTGCCCTCTTTTACC	GAAGCCGTCTTGCATCGTGAT
GPR84	GTGCTGGGCTATCGTTATGTT	GAATCGGGTACGGAGCTTGG
β-ACTIN	CGTGGGCCGCCCTAGGCACCA	TTGGCTTAGGGTTCAGGGGGG

### Identification of miRNAs targeting genes in our diagnostic models

Perform miRNA target prediction analysis to identify miRNAs that target genes in our diagnostic model by binding to their 3′ UTRs with four online public websites, including TargetScan [14], mirDIP [15], miRWalk [16], and miRmap [17] databases. Subsequently, target miRNAs predicted concordantly by the four databases were chosen to construct a miRNA-mRNA network by Cytoscape software [18].

### Statistical analysis

Categorical data were compared with the Pearson chi-square test or Fisher exact test whenever appropriate, and quantitative variables were analyzed using the independent-sample *t*-test. The clinical characteristics of 159 infants are shown in Table 2. Results were considered statistically significant when the *p* value was < 0.05.

**Table 2:** Clinical characteristics of HCC patients involved in the study

Characteristics	Normal infants (*N* = 99)	EOS infants (*N* = 60)	*p* value
Gender, *n* (%)			
Male	56 (56.5)	32 (53.3)	0.743
Female	43 (43.4)	28 (46.6)	
Age, day, mean ± SD	4 ± 2.0	4 ± 1.9	0.288
PCT, ng/mL, mean ± SD	9.39 ± 27.63	22.67 ± 36.71	0.002
Hb, g/L, mean ± SD	117.69 ± 31.56	100.20 ± 22.18	0.002
NEU%, mean ± SD	56.51 ± 18.60	51.23 ± 18.60	0.282
CRP, mg/L, mean ± SD	23.36 ± 41.27	43.16 ± 61.18	0.001

## Results

### Identification of overlapped DEGs

**Fig. 1:**
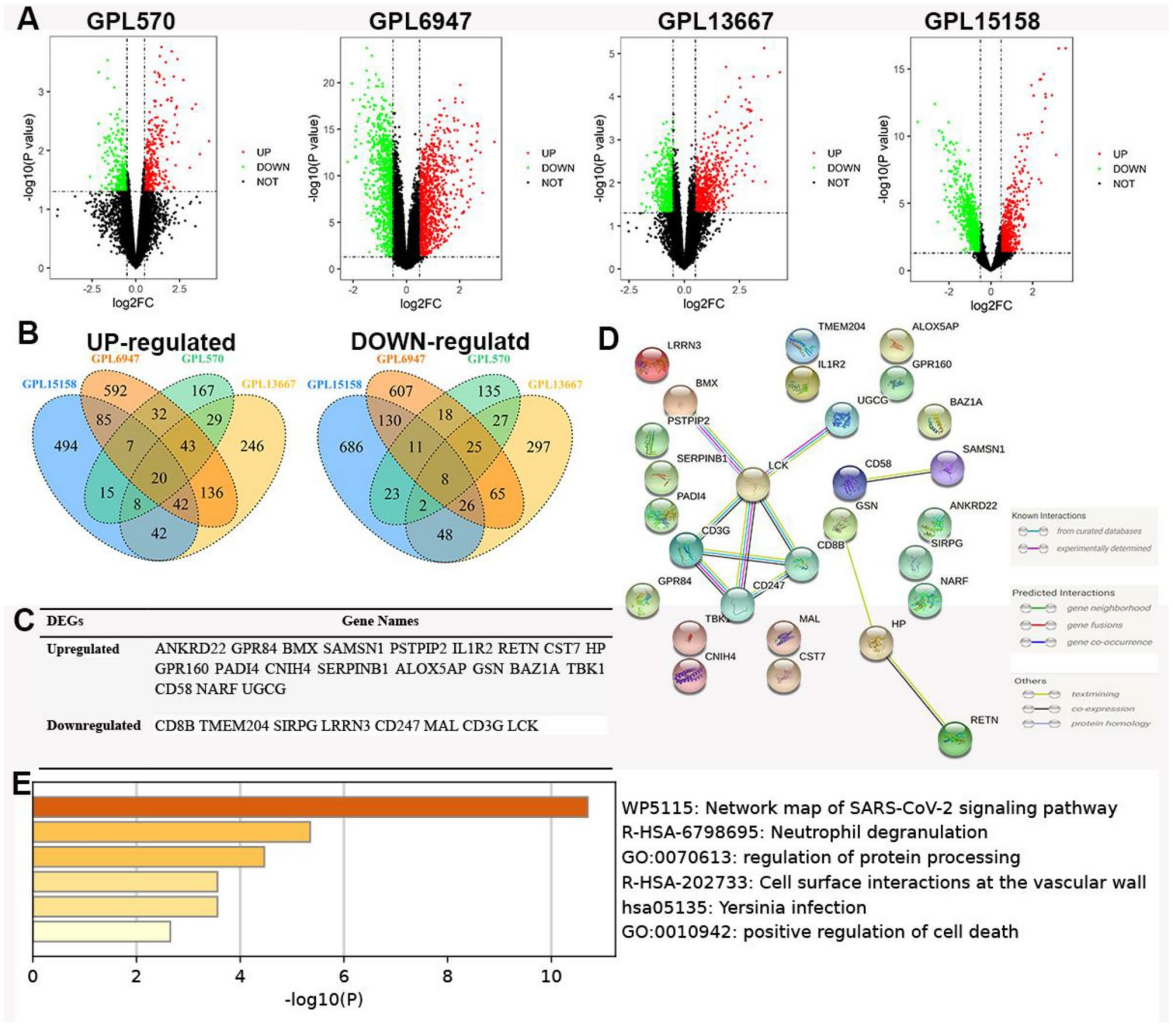
Identification of the common DEGs on the four platforms in the GSE25504 dataset. **A** Volcano plots of DEGs on four platforms. **B** The 28 duplicated DEGs were changed in EOS samples. **C** Names of the 28 overlapping DEGs. **D** PPI network construction. **E** Functional enrichment analysis

As shown in Fig. 1A, DEGs on the four platforms were screened with a cut-off criterion of *p* value < 0.05 and |logFC|> 0.5; 20 upregulated genes and 8 down-regulated genes were identified as common DEGs (Fig. 1B), which are shown in Fig. 1C. We also constructed a protein–protein interaction (PPI) network based on these DEGs (Fig. 1D). Twenty-eight nodes and 11 edges were acquired from the PPI network. The local clustering coefficient was 0.332, and the PPI enrichment *p* value was equal to 4.78*e* − 05. We found that CD247, CD3G, CD8B, and LCK might be the core genes. Finally, the results of the GSEA analysis showed that these overlapping DEGs are mainly associated with pathways related to infection, neutrophil degranulation, and cellular immune response (Fig. 1E).

### Identification of critical genes involved in neonatal EOS

**Fig. 2:**
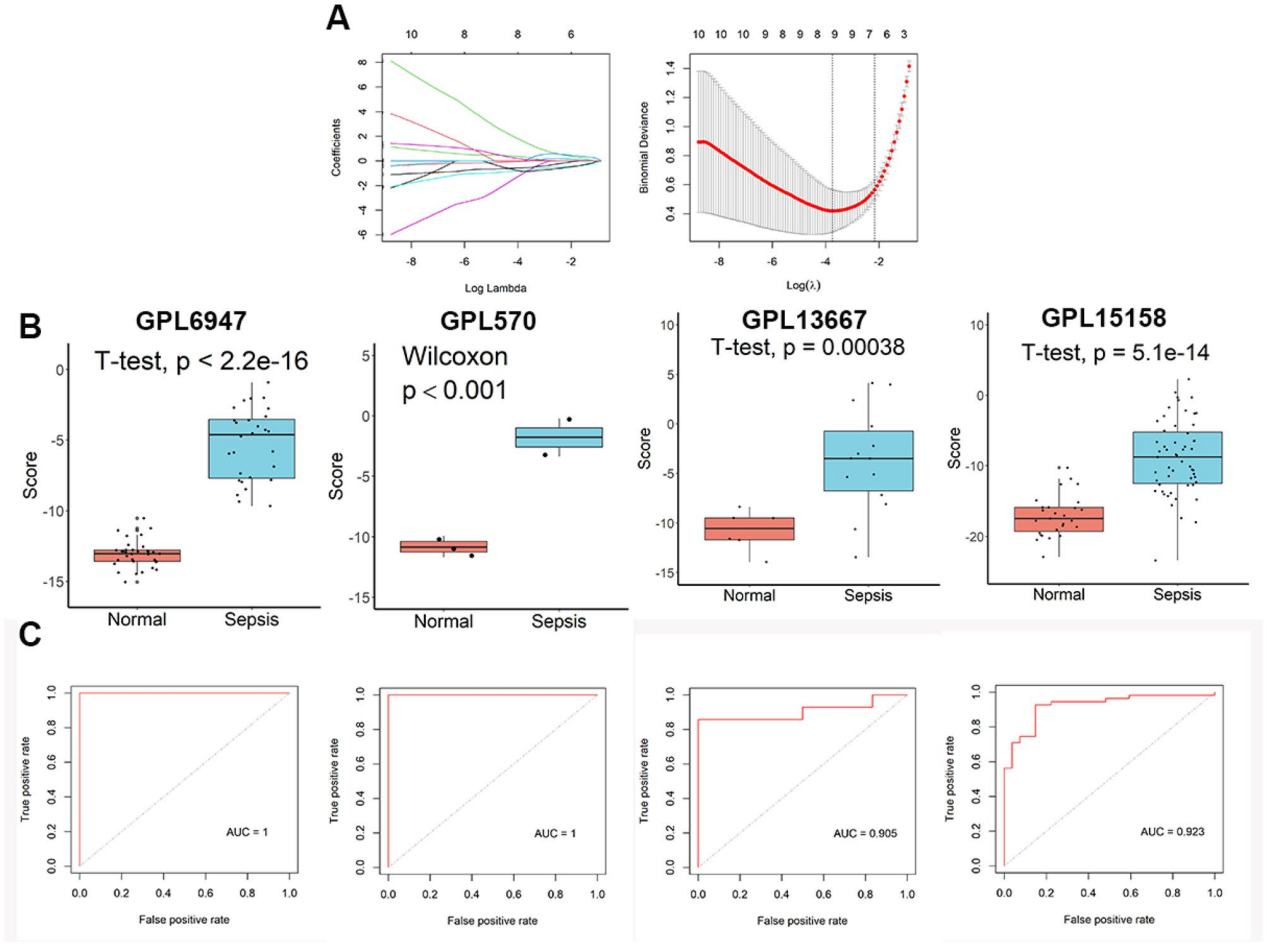
Construction and ROC analysis of this eight-gene diagnostic model in the GSE25504 dataset. **A** Adjustment of parameter selection in binomial LASSO models by 10 times cross-validation. **B** Difference analysis of scores between normal and EOS infants. **C** ROC analysis of the eight-gene diagnostic model

According to the binomial LASSO analysis, when *λ*_*min*_ was equal to 0.02353066, eight genes (CST7, CD3G, CD8B, CD247, SIRPG, GPR84, MAL, and ANKRD22) were identified that most accurately predicted EOS (Fig. 2A). *Score* = (1.54278655 × CST7) − (0.02890854 × cd247) − (0.78324710 × CD3G) − (0.95299570 × CD8B) − (0.88133909 × SIRPG) + (0.33541335 × GPR84) + 0.11719602 × ANKRD22) − (0.55729858 × MAL). As shown in Fig. 2B, infants with EOS had significantly higher scores than these normal infants on the four platforms. What is more, the characterization of this eight-genes signature showed good diagnostic power with AUCs of 1, 1, 0.905, and 0.923 on the four platforms, respectively (Fig. 2C).

### Verification of the diagnostic model in a clinical cohort

**Fig. 3:**
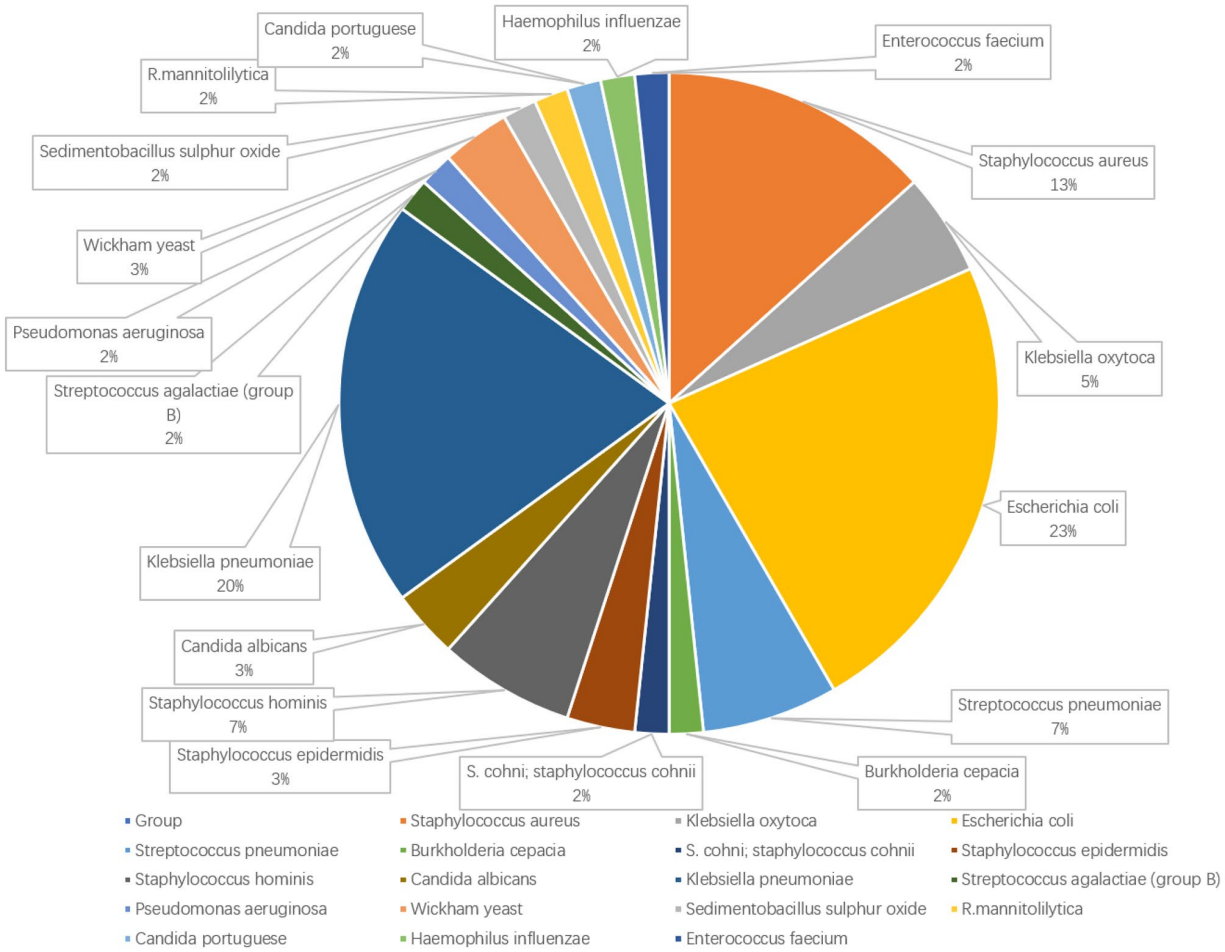
Types of bacteria that infected EOS infants

**Fig. 4:**
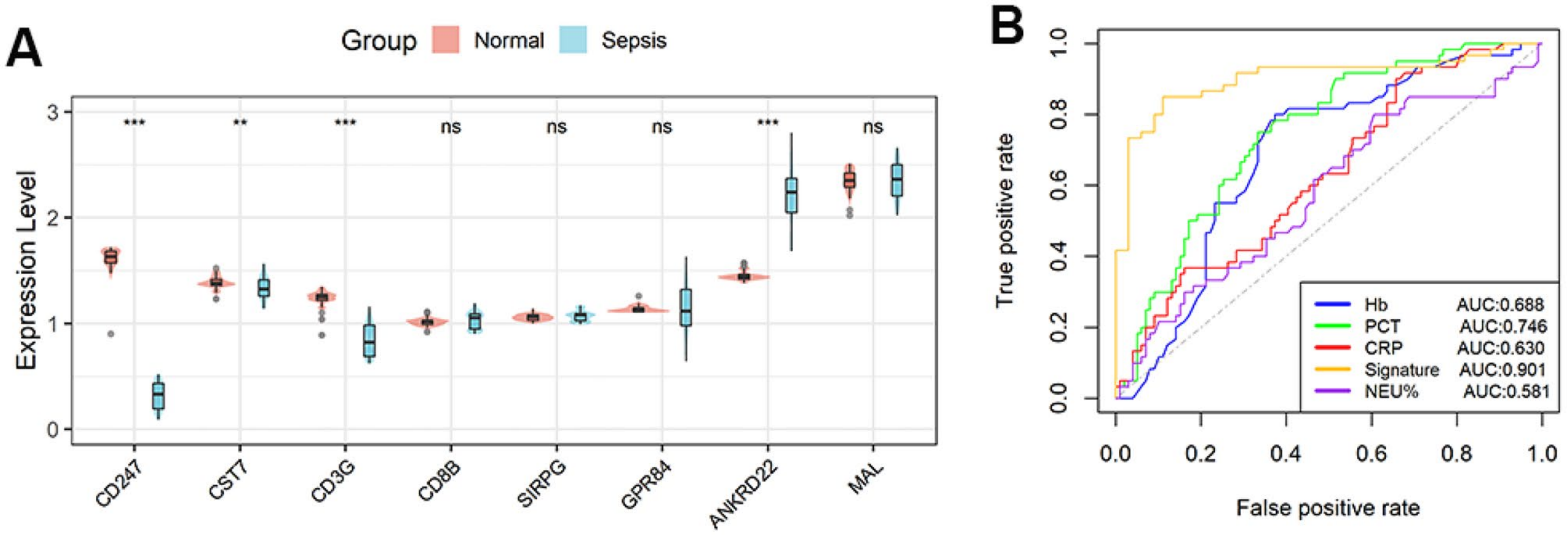
Verification of this eight-gene diagnostic model in a clinical dataset. **A** Difference analysis of eight genes expression between normal and EOS infants. **B** ROC analysis of the diagnostic model and other conventional inflammatory indicators in the clinical cohort. ns, not significant; **p < 0.01; ***p < 0.001

To verify the capability of the model in the diagnosing of neonatal EOS in actual clinical practice, qRT-PCR analysis was performed in a clinical cohort. There were no differences in gender, age, and NEU% levels between the EOS and normal infant groups (Table 2). The types of bacteria that the EOS infants were infected with are shown in Fig. 3. EOS infants had higher levels of PCT and CRP and lower levels of Hb when compared with those in the normal group. All genes in the model except CD8B, SIRPG, GPR84, and MAL were differentially expressed in the peripheral blood of EOS infants and normal infants (Fig. 4A). Four genes that were not significantly different were not included when calculating infant scores using the same formula. ROC analysis revealed that this diagnostic model performed well in our clinical cohort (Fig. 4B). In addition, when compared with conventional inflammatory indicators such as C-reactive protein (CRP), hemoglobin (Hb), neutrophil percentage (NEU%), and procalcitonin (PCT), the model has better diagnostic performance.

### Construction of the miRNA-mRNA network

**Fig. 5:**
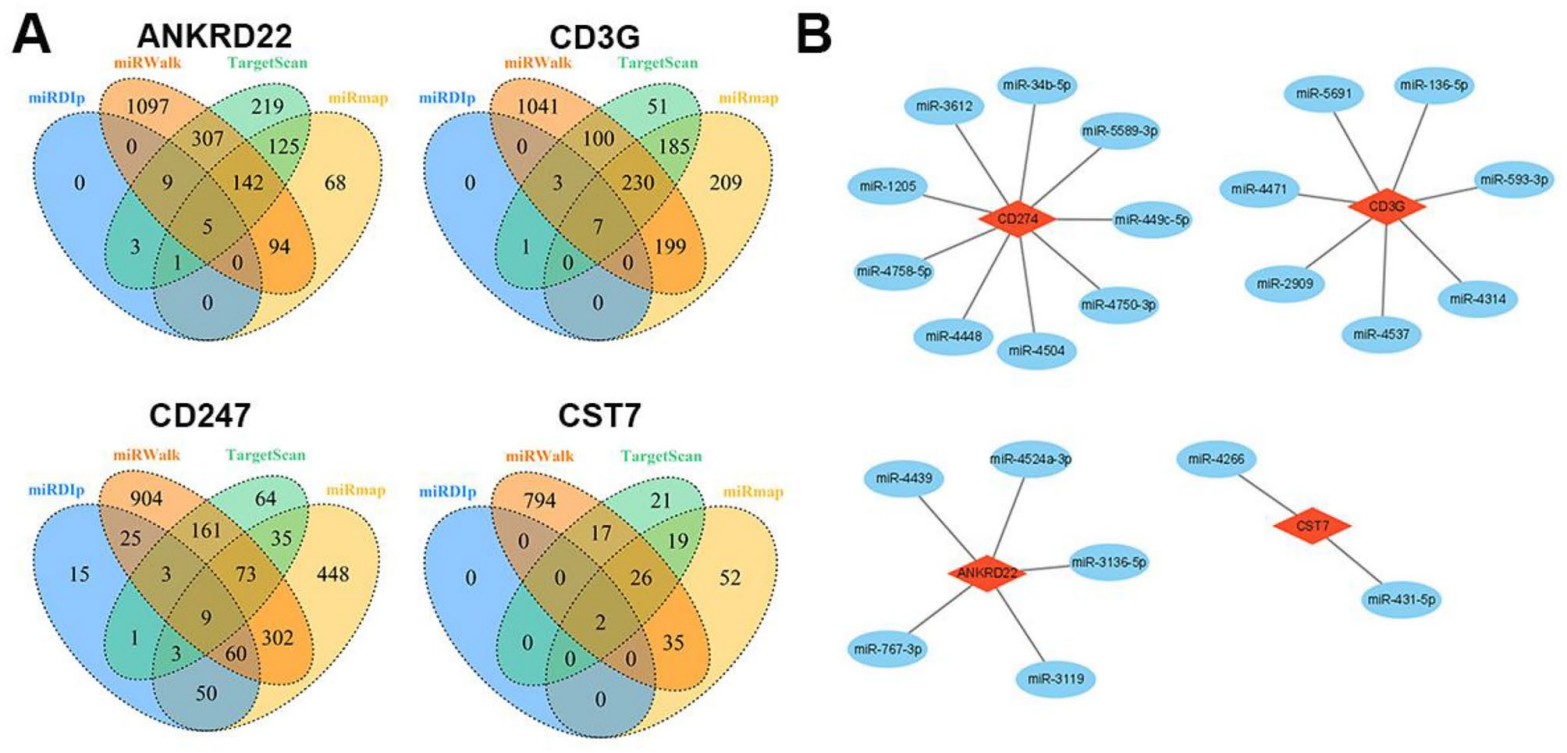
Construction of miRNA-mRNA network. **A** miRNA target prediction analysis within the four online public websites. **B** The miRNA-mRNA network consists of genes in our diagnostic model and potential target miRNAs

Due to the results of miRNA target prediction analysis within the four online public websites, as shown in Fig. 5A, a total of 5 miRNAs targeting the 3′ UTR of ANKRD22, 7 miRNAs targeting the 3′ UTR of CD3G, 2 miRNAs targeting the 3′ UTR of CST7, and 9 miRNAs targeting the 3′ UTR of CD247 were identified. Then, the miRNA-mRNA network consisted of genes in our diagnostic model, and potential target miRNAs were constructed (Fig. 5B).

## Discussion

Despite breakthroughs in prenatal care and antibiotic prophylaxis, neonatal EOS, unfortunately, remains the third leading cause of neonatal death worldwide, due to a lack of reliability in identifying those infants who are infected [19]. Blood cultures remain the current gold standard for the diagnosis of EOS, however, its sensitivity is low in neonates, and diagnosis is delayed [20]. As a result, many newborns with suspected EOS, especially premature infants, are routinely treated with broad-spectrum intravenous antibiotics for several days, although empirical antibiotic administration may have a potential negative impact on the growth and development of newborns [9]. Hence, to minimize unnecessary antibiotic exposure in newborns with suspected EOS, there is an urgent need in clinical neonatal management for a more reliable method to diagnose EOS that utilizes neonatal blood or tissue.

In our current bioinformatics analysis study, potential diagnostic biomarkers between the sepsis and control groups were identified on the four platforms of the GSE25504 dataset. Then, we selected 28 common DEGs to construct a binomial LASSO model. Finally, a prognostic model consisting of eight genes was built and showed good diagnostic power on the four platforms. To verify the capability of the model in the diagnosing of neonatal EOS in actual clinical practice, qRT-PCR analysis was performed in a clinical cohort that consisted of peripheral blood samples from 99 normal and 60 EOS infants. All genes in the model except CD8B, SIRPG, GPR84, and MAL were differentially expressed in the peripheral blood of EOS infants and normal infants. After the score of infants was calculated with the same formula based on the four DEGs (CST7, CD3G, CD247, and ANKRD22), ROC analysis revealed that this diagnostic model performed well in our clinical cohort. In addition, when compared with conventional inflammatory indicators such as CRP, Hb, NEU%, and PCT, the model has better diagnostic performance. All the above results indicated that the diagnostic model constructed in our study could separate EOS infants from normal infants.

Most of the four genes in our diagnostic model are more or less associated with sepsis. Neutrophil-specific CST7 was significantly upregulated in the whole blood of patients with sepsis, and its encoded Cysteine F was involved in regulating the cytotoxicity of natural killer (NK) cells within the tumor microenvironment [21, 22]. CD3G is an upregulated gene involved in T cells and has been reported to be inversely correlated with sequential organ failure (SOFA) and mortality in sepsis [23]. CD247 has been reported to be involved in human and murine sepsis by many studies, and it can be involved in the occurrence and development of sepsis as a key gene of sepsis [24–29]. As for homo sapiens ankyrin repeat domain 22 (ANKRD22), which encoded a specific mitochondrial protein, it has been demonstrated to be involved in the progression of multiple tumors, including colorectal cancer [30], breast cancer [31], pancreatic cancer [32], prostate cancer [33], and nonsmall-cell lung cancer [34]; however, there are few relevant studies on sepsis and further investigation is required.

When compared with the previous study, in which miRNAs obtained from umbilical cord plasma or umbilical cord tissue could well distinguish neonatal EOS from normal infants [35], this four-gene diagnostic model has a better discriminatory ability. In addition, the umbilical cord plasma or tissue may no longer be readily available when the newborn presents with signs of sepsis, making our model more practical. There is no doubt that our study has some limitations. The individual and geographic variability of EOS infants may affect the performance of this model. In addition, the small sample size in our clinical cohort limits the validation of the model, and future multicenter randomized controlled studies are needed to evaluate this model. Finally, our study did not include blood-culture-negative infants with EOS. Considering that blood-culture-positive and negative infants may have different peripheral blood transcriptome genetic changes, we need to collect blood-culture-negative infants with EOS and detect the expression changes of the four genes in their peripheral blood to determine whether the four-gene signature we constructed could identify septic infants with negative blood cultures in the future.

## Conclusions

In summary, we constructed a four-gene diagnostic model that can accurately differentiate neonatal EOS with bacterial infection by bioinformatics analysis, which can be used as an ancillary test for the diagnosis of neonatal EOS with bacterial infection in the future.

## Data Availability

The datasets used and/or analyzed during the current study are available from the corresponding author upon reasonable request.

## Abbreviations

EOS: Early-onset sepsis
AKI: Acute kidney injury
NEC: Necrotizing enterocolitis
ESR: Erythrocyte sedimentation rate
PCT: Procalcitonin
CRP: C-reactive protein
DEGs: Differentially expressed genes
GEO: Gene expression omnibus
GSEA: Gene set enrichment analysis
LASSO: Least absolute shrink and selection operator
ROC: Receiver operating characteristic curve
qRT-PCR: Quantitative real-time PCR
PBMCs: Peripheral blood mononuclear cells
PPI: Protein-protein interaction
NEU: Neutrophil
Hb: Hemoglobin
NK: Natural killer
SOFA: Sequential organ failure
ANKRD22: Homo sapiens ankyrin repeat domain 22

## References

[1] Singer M, Deutschman CS, Seymour CW, Shankar-Hari M, Annane D, Bauer M, Bellomo R, Bernard GR, Chiche JD, Coopersmith CM (2016). The Third International Consensus Definitions for Sepsis and Septic Shock (Sepsis-3). JAMA.

[2] Bagshaw SM, George C, Bellomo R (2008). Early acute kidney injury and sepsis: a multicentre evaluation. Crit Care.

[3] Weitkamp JH (2020) The role of biomarkers in suspected neonatal sepsis. Clin Infect Dis 10.1093/cid/ciaa869 32881996

[4] Schrag SJ, Farley MM, Petit S, Reingold A, Weston EJ, Pondo T, Hudson Jain J, Lynfield R (2016) Epidemiology of invasive early-onset neonatal sepsis, 2005 to 2014. Pediatrics 138(6) 10.1542/peds.2016-2013 27940705

[5] Kuppala VS, Meinzen-Derr J, Morrow AL, Schibler KR (2011). Prolonged initial empirical antibiotic treatment is associated with adverse outcomes in premature infants. J Pediatr.

[6] Cho I, Yamanishi S, Cox L, Methé BA, Zavadil J, Li K, Gao Z, Mahana D, Raju K, Teitler I (2012). Antibiotics in early life alter the murine colonic microbiome and adiposity. Nature.

[7] Ting JY, Synnes A, Roberts A, Deshpandey A, Dow K, Yoon EW, Lee KS, Dobson S, Lee SK, Shah PS (2016). Association between antibiotic use and neonatal mortality and morbidities in very low-birth-weight infants without culture-proven sepsis or necrotizing enterocolitis. JAMA Pediatr.

[8] Flannery DD, Ross RK, Mukhopadhyay S, Tribble AC, Puopolo KM, Gerber JS (2018). Temporal trends and center variation in early antibiotic use among premature infants. JAMA Netw Open.

[9] Kuzniewicz MW, Puopolo KM (2020). Antibiotic stewardship for early-onset sepsis. Semin Perinatol.

[10] Lin GC, Küng E, Smajlhodzic M, Domazet S, Friedl HP, Angerer J, Wisgrill L, Berger A, Bingle L, Peham JR et al (2021) Directed transport of CRP across in vitro models of the blood-saliva barrier strengthens the feasibility of salivary CRP as biomarker for neonatal sepsis. Pharmaceutics 13(2) 10.3390/pharmaceutics13020256 PMC7917918 33673378

[11] Honore PM, Redant S, Kaefer K, Barreto Gutierrez L, Kugener L, Attou R, Gallerani A, De Bels D (2021) Procalcitonin is useful for antibiotic deescalation in sepsis and septic shock: beware of some confounders! Crit Care Med 49(6):e659 10.1097/CCM.0000000000004934 34011841

[12] Zhou Y, Zhou B, Pache L, Chang M, Khodabakhshi AH, Tanaseichuk O, Benner C, Chanda SK (2019). Metascape provides a biologist-oriented resource for the analysis of systems-level datasets. Nat Commun.

[13] Zhang G (2020). Expression and prognostic significance of BANF1 in triple-negative breast cancer. Cancer Manag Res.

[14] Lewis BP, Burge CB, Bartel DP (2005). Conserved seed pairing, often flanked by adenosines, indicates that thousands of human genes are microRNA targets. Cell.

[15] Tokar T, Pastrello C, Rossos AEM, Abovsky M, Hauschild AC, Tsay M, Lu R, Jurisica I (2018) mirDIP 4.1-integrative database of human microRNA target predictions. Nucleic Acids Res 46(D1):D360-d370 10.1093/nar/gkx1144 PMC5753284 29194489

[16] Sticht C, De La Torre C, Parveen A, Gretz N (2018). miRWalk: an online resource for prediction of microRNA binding sites. PLoS ONE.

[17] Vejnar CE, Blum M, Zdobnov EM (2013) miRmap web: comprehensive microRNA target prediction online. Nucleic Acids Res 41:W165–168 10.1093/nar/gkt430 PMC3692044 23716633

[18] Shannon P, Markiel A, Ozier O, Baliga NS, Wang JT, Ramage D, Amin N, Schwikowski B, Ideker T (2003). Cytoscape: a software environment for integrated models of biomolecular interaction networks. Genome Res.

[19] Liu L, Oza S, Hogan D, Perin J, Rudan I, Lawn JE, Cousens S, Mathers C, Black RE (2015). Global, regional, and national causes of child mortality in 2000–13, with projections to inform post-2015 priorities: an updated systematic analysis. Lancet.

[20] Mithal LB, Palac HL, Yogev R, Ernst LM, Mestan KK (2017). Cord blood acute phase reactants predict early onset neonatal sepsis in preterm infants. PLoS ONE.

[21] Sawyer AJ, Garand M, Chaussabel D, Feng CG (2021). Transcriptomic profiling identifies neutrophil-specific upregulation of cystatin F as a marker of acute inflammation in humans. Front Immunol.

[22] Perišić Nanut M, Sabotič J, Švajger U, Jewett A, Kos J (2017). Cystatin F affects natural killer cell cytotoxicity. Front Immunol.

[23] Almansa R, Heredia-Rodríguez M, Gomez-Sanchez E, Andaluz-Ojeda D, Iglesias V, Rico L, Ortega A, Gomez-Pesquera E, Liu P, Aragón M (2015). Transcriptomic correlates of organ failure extent in sepsis. J Infect.

[24] Zeng X, Feng J, Yang Y, Zhao R, Yu Q, Qin H, Wei L, Ji P, Li H, Wu Z (2021). Screening of key genes of sepsis and septic shock using bioinformatics analysis. J Inflamm Res.

[25] Gong FC, Ji R, Wang YM, Yang ZT, Chen Y, Mao EQ, Chen EZ (2020). Identification of potential biomarkers and immune features of sepsis using bioinformatics analysis. Mediators Inflamm.

[26] Liang J, Wu W, Wang X, Wu W, Chen S, Jiang M (2022). Analysis of sepsis markers and pathogenesis based on gene differential expression and protein interaction network. Journal of healthcare engineering.

[27] Kim KS, Jekarl DW, Yoo J, Lee S, Kim M, Kim Y (2021). Immune gene expression networks in sepsis: a network biology approach. PLoS ONE.

[28] Chen M, Chen X, Hu Y, Cai X (2020) Screening of key genes related to the prognosis of mouse sepsis. Biosci rep 40(10) 10.1042/BSR20202649 PMC7601352 33015708

[29] Chen J, Lin M, Zhang S (2019). Identification of key miRNA-mRNA pairs in septic mice by bioinformatics analysis. Mol Med Rep.

[30] Pan T, Liu J, Xu S, Yu Q, Wang H, Sun H, Wu J, Zhu Y, Zhou J, Zhu Y (2020). ANKRD22, a novel tumor microenvironment-induced mitochondrial protein promotes metabolic reprogramming of colorectal cancer cells. Theranostics.

[31] Wu Y, Liu H, Gong Y, Zhang B, Chen W (2021). ANKRD22 enhances breast cancer cell malignancy by activating the Wnt/β-catenin pathway via modulating NuSAP1 expression. Bosn J Basic Med Sci.

[32] Luo L, Li Y, Huang C, Lin Y, Su Y, Cen H, Chen Y, Peng S, Ren T, Xie R (2021). A new 7-gene survival score assay for pancreatic cancer patient prognosis prediction. Am J Cancer Res.

[33] Qiu Y, Yang S, Pan T, Yu L, Liu J, Zhu Y, Wang H (2019). ANKRD22 is involved in the progression of prostate cancer. Oncol Lett.

[34] Yin J, Fu W, Dai L, Jiang Z, Liao H, Chen W, Pan L, Zhao J (2017). ANKRD22 promotes progression of non-small cell lung cancer through transcriptional up-regulation of E2F1. Sci Rep.

[35] Ernst LM, Mithal LB, Mestan K, Wang V, Mangold KA, Freedman A, Das S (2021). Umbilical cord miRNAs to predict neonatal early onset sepsis. PLoS ONE.

